# Does gibberellin biosynthesis play a critical role in the growth of *Lolium perenne*? Evidence from a transcriptional analysis of gibberellin and carbohydrate metabolic genes after defoliation

**DOI:** 10.3389/fpls.2015.00944

**Published:** 2015-11-03

**Authors:** Qianhe Liu, Chris S. Jones, Anthony J. Parsons, Hong Xue, Susanne Rasmussen

**Affiliations:** ^1^Forage Improvement, Grasslands Institute, AgResearch Ltd.Palmerston North, New Zealand; ^2^Institute of Agriculture and Environment, Massey UniversityPalmerston North, New Zealand

**Keywords:** perennial ryegrass, defoliation, gibberellins, carbohydrates, gene expression, fructans

## Abstract

Global meat and milk production depends to a large extent on grazed pastures, with *Lolium perenne* being the major forage grass in temperate regions. Defoliation and subsequent regrowth of leaf blades is a major and essential event with respect to *L. perenne* growth and productivity. Following defoliation, carbohydrates (mainly fructans and sucrose) have to be mobilized from heterotrophic tissues to provide energy and carbon for regrowth of photosynthetic tissues. This mobilization of reserve carbohydrates requires a substantial change in the expression of genes coding for enzymes involved in carbohydrate metabolism. Here we tested the hypothesis that gibberellins (GA) are at the core of the processes regulating the expression of these genes. Thus, we examined the transcript profiles of genes involved in carbohydrate and GA metabolic pathways across a time course regrowth experiment. Our results show that following defoliation, the immediate reduction of carbohydrate concentrations in growing tissues is associated with a concomitant increase in the expression of genes encoding carbohydrate mobilizing invertases, and was also associated with a strong decrease in the expression of fructan synthesizing fructosyltransferase genes. We also show that the decrease in fructan levels is preceded by increased expression of the GA activating gene *GA*_3_-*oxidase* and decreased expression of the GA inactivating gene *GA*_2_*-oxidase* in sheaths. *GA*_3_-*oxidase* expression was negatively, while *GA*_2_*-oxidase* positively linked to sucrose concentrations. This study provides indicative evidence that gibberellins might play a role in *L. perenne* regrowth following defoliation and we hypothesize that there is a link between gibberellin regulation and sugar metabolism in *L. perenne*.

## Introduction

The global consumption of animal derived food (meat and milk) is projected to increase considerably in the coming decades, driven mainly by population growth, economic growth and urbanization in China and Southeast Asia (FAO, 2011). Currently, more than 60% of the global meat production and 90% of milk production is produced either exclusively on grazed pastures, or in mixed systems with a large proportion of grazed and conserved (silage) pasture. This high demand for pasture dry matter production is met by increased intensification with a concomitant increase in, mainly nitrogen (N), inputs. These additional inputs come at a high cost to the environment due to high levels of N losses either leached as nitrate and polluting waterways, or emitted from soils as nitrous oxide, a greenhouse gas almost 300 times more potent than carbon dioxide (Kebreab et al., 2001; Tas, 2007; Ellis et al., 2011; van Groenigen et al., 2011). The root cause of these high N losses from grazed pastures is the discrepancy between the relatively low ruminants' dietary N requirements for optimal performance (around 2.5% N) and the plants' requirements for very high N concentrations in photosynthetic tissues (4.5–5% N) for maximal photosynthetic capacity (Van Soest, 1982; Woledge and Pearse, 1985). Overcoming this dilemma would require the development of plants with an improved ability to grow at low N supply and a higher C/N ratio in photosynthetic active tissues, and we proposed previously that a prerequisite for this might be to change a plant's growth “strategy” (Parsons et al., 2011, 2013a,b).

Perennial ryegrass (*Lolium perenne* L.) is a cool-season grass and the main pasture plant in temperate zones such as Northern Europe and New Zealand (Parsons and Chapman, 2000). So far, breeding has made little progress in improving ryegrass yield at low N inputs (Parsons et al., 2011). Heterotrophic tissues in the meristematic cell division zone and the enclosed elongation zone of immature blades of this grass species generally depend on the supply of newly fixed carbon from autotrophic mature blades to support growth (Morvan-Bertrand et al., 1999; Parsons et al., 2013a). Regular defoliation of ryegrass by grazing animals or mowing removes most of the photosynthetic tissues capable of fixing CO_2_ and deprives the growing tissues of sugars. This depletion of sugars induces a shift from carbon storage [mainly high molecular weight (HMW) fructans in ryegrass] to low molecular weight (LMW) sugars to support rapid elongation of enclosed immature blades to form new photosynthetic tissue, a prerequisite for continued plant growth (Schnyder et al., 1990; Morvan-Bertrand et al., 1999, 2001; Schnyder and de Visser, 1999). These processes require a substantial change in the expression of genes encoding enzymes involved in carbohydrate mobilization and accumulation (Morvan-Bertrand et al., 1999, 2001; Lee et al., 2011). Sucrose mobilization is catalyzed by the action of invertases, which are enzymes that cleave sucrose into glucose and fructose, and different forms of invertases, including cell wall (CWInv), cytoplasmic (CytInv), and vacuolar invertase (VacInv), are distributed between cellular compartments (Kingston-Smith et al., 1999; Cairns and Gallagher, 2004). Fructan mobilization is catalyzed by fructan exohydrolases (FEHs), in *L. perenne* two FEHs have been characterized so far, 1-FEH (Lothier et al., 2007) and 6-FEH (Lothier et al., 2014).

Plant hormones have been shown to regulate changes from the accumulation to mobilization of carbohydrates, and *vice versa* (Morvan et al., 1997; Perata et al., 1997; Morvan-Bertrand et al., 2001; Gibson, 2004; Ranwala and Miller, 2008; Eveland and Jackson, 2012; Matsoukas, 2014). The important role of one of these classes of hormones, the gibberellins (GAs), for vegetative growth in cereals is obvious by the success of the “green revolution” which produced wheat, rice and other cereal cultivars with stunted growth beneficial for grain production by selecting mutants in the GA biosynthetic pathway or impaired in GA reception (Peng et al., 1999). GAs are plant hormones that promote stem and leaf growth and also activate dormant enzyme systems (see reviews by Yamaguchi, 2008; Colebrook et al., 2014). *GA*_3_*-oxidase* and *GA*_20_*-oxidase* are both genes encoding enzymes which activate gibberellin precursors in the GA biosynthetic pathway, while *GA*_2_*-oxidase* codes for a GA-inactivating enzyme (Yamaguchi, 2008), while DELLA is a protein that negatively regulates plant growth (Achard and Genschik, 2009). The role of GAs as mediators of environmental stimuli is well established (Gocal et al., 1999; MacMillan et al., 2005; Yamaguchi, 2008) and a number of reports have indicated that the GA-mediated elongation of shoots in various plants occurs as a result of an increase in cell division (Sauter et al., 1995) and cell elongation (Smith et al., 1996; Matsukura et al., 1998). Gibberellins are regulated by nutrient levels, particularly low soluble sugar levels appear to activate and/or induce GAs; these activated GAs subsequently induce the expression of a range of hydrolases involved in the enzymatic degradation and mobilization of storage macromolecules, for example starch and proteins to provide substrates for growth (Bewley and Black, 1985; Gibson, 2004; Hong et al., 2012; Paparelli et al., 2013).

Active GA_3_ has been demonstrated to be present in wheat (*Triticum aestivum*) and other cereals, but it is absent from the vegetative tissues of the dicots *Arabidopsis thaliana* and pea (*Pisum sativum*; Appleford et al., 2006), indicating that the types of active GAs vary in plant species and may respond to altering environmental stimuli (e.g., defoliation) differentially. Morvan et al. (1997) showed that defoliation induces a strong and rapid increase in fructan exohydrolase (FEH, EC 3.2.1.80) activity and a reduction of fructans in the immature elongating leaf bases of *L. perenne*. The increased activity of FEH was strongly inhibited by an inhibitor of GA biosynthesis, uniconazole, and this effect was reversed by treatment with active GA_3_, suggesting that the increased FEH activity following defoliation was mediated by the biosynthesis of GA_3_. However, neither in a subsequent study by the same researchers (Morvan-Bertrand et al., 2001), nor, to our knowledge, in any other studies has GA_3_ ever been reported to be present in *L. perenne*, even though it has been shown to induce stem and leaf elongation when exogenously applied (MacMillan et al., 2005; Parsons et al., 2013a). The above evidence indicates that GAs may be core to regulating vegetative growth of *L. perenne* in a tight interplay with carbohydrate metabolism, but little other information is available about the situation in forage grasses. Since GA biosynthesis is regulated by GA-related genes (see reviews by Yamaguchi, 2008; Colebrook et al., 2014), it is worth pursuing further evidence to test if GA and carbohydrate associated genes are differentially regulated during regrowth following defoliation in *L. perenne*. In an earlier experiment using exogenous gibberellin (GA_3_) we demonstrated that it is possible to increase the growth of *L. perenne* without increasing N supply and we suggested that GA_3_ might be at the core of limiting ryegrass growth at a given N availability (Parsons et al., 2013a). Here, we undertook to investigate a possible role of GA biosynthesis during regrowth of perennial ryegrass, and report on a time-course experiment in which carbohydrate concentrations in plant tissues and transcript profiles of sugar- and GA-related genes were analyzed during plant regrowth following defoliation.

## Materials and methods

### Plant material

Seeds of the *L. perenne* cultivar “Expo” (PGG Wrightson Seeds Ltd., NZ) were germinated and grown for 2 months in 9 × 9 × 18 (d × w × h) cm pots (one seedling per pot) containing commercial potting mixture (Midland Horticulture, Palmerston North NZ) before the study commenced. Plants were randomly distributed and grown in a glasshouse with supplementary lighting and heating (~17–22°C at day; 9–13°C at night) to ensure good plant growth. During this pre-study period, plants were cut back every 4 weeks to 6 cm above soil level and watered every second day to “pot field capacity.” No additional nutrients were applied during the period of the experiment.

**Figure 1:**
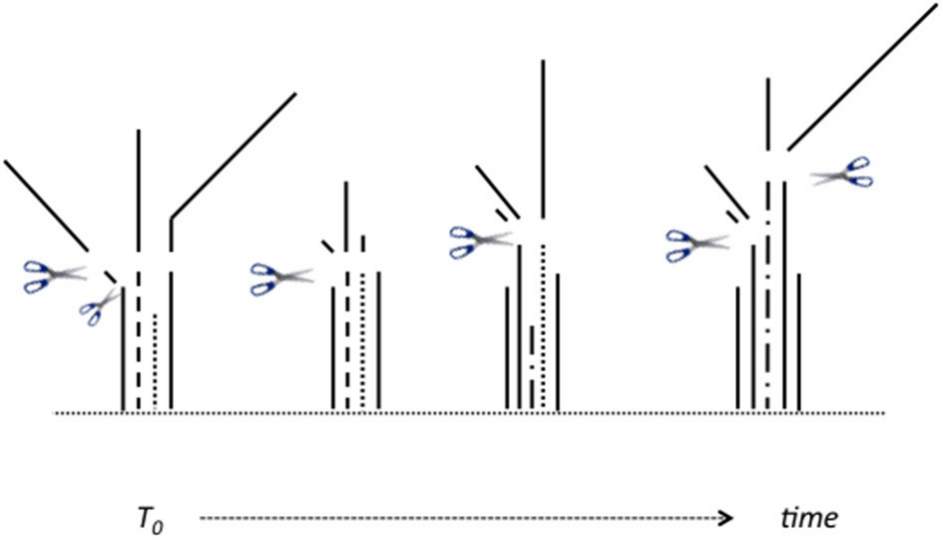
**Schematic of how harvested plant parts were allocated to three ***categories of tissues***, as plants regrew following defoliation**. Tissues were divided between: *emerged* leaf tissues (“EM”); *enclosed and elongating* leaf tissues (“EE”), and *sheaths* (“S”) in text and graphs. Emerged tissue “EM” refers to all tissues removed above the initial 6 cm cutting height (e.g., at T0), and subsequently all emerged tissues either above that same height, or above/beyond the ligule of any ligulated leaves, as these became present (see solid lines above/beyond scissor icons). The fully enclosed bases of unligulated, and still elongating, leaves (“EE,” see broken lines in Figure) were dissected out from the enclosing older sheaths. The remaining tissue is sheath, “S,” being the non-elongating bases of older ligulated leaves (see solid lines below scissor icons). See Materials and Methods for further explanation/basis for this approach.

The critical experiment commenced in July 2010. Two-month old plants (40 plants in total) were cut back to 6 cm above soil level, which removed nearly all emerged leaf material, see Figure 1. At this first time point, T0, and on seven subsequent occasions after 2, 4, 24, 48, 72, 168, and 336 h (h) regrowth, five individual plants (as experimental replicates) were harvested at the soil level; dead tissues removed; and plant shoots dissected into three *categories* of tissues (Figure 1): sheath tissues (S), elongating enclosed leaf tissues (EE), and emerged leaf tissue (EM). The latter included all tissue that had emerged beyond the (initially) cutting level, or (later) all tissue that had emerged beyond the ligule of the youngest ligulated leaf, if one was present. Plants harvested at 0 h represent “before defoliation controls.” The harvested tissues were immediately flash-frozen and ground in a pestle and mortar under liquid nitrogen. A portion of the powdered samples was freeze-dried for carbohydrate analysis, and the rest stored at −80°C for subsequent RNA isolation.

### Water soluble carbohydrate analysis

Water soluble carbohydrates (WSCs) were extracted and quantified as described previously (Hunt et al., 2005). Powdered, freeze-dried plant material (25 mg) was extracted with 2 ml 80% ethanol for the LMW WSC fraction and subsequently with 2 ml water for the HMW WSC fraction. The ethanol extracted LMW WSC fraction contains a mixture of glucose, fructose, sucrose and low DP (degree of polymerization) fructans and the water extracted HMW WSC fraction contains mainly high DP fructans (Rasmussen et al., 2008). Extracts were briefly centrifuged and WSCs determined in the supernatants using anthrone as a colorimetric reagent as described previously (Jermyn, 1956). Glucose, fructose, and sucrose concentrations were determined enzymatically in the LMW WSC extracts as described (Rasmussen et al., 2007).

### Isolation of GA-related genes from *L. perenne*

During this study, we isolated two full length *L. perenne* mRNA sequences encoding candidates for a GA_3_-oxidase (Lp*GA3ox*) and a key growth regulator, DELLA (Lp*DELLA*). The corresponding gene sequences have been deposited in NCBI Genebank as accession numbers KP954695 (Lp*GA3ox*) and KP954694 (Lp*DELLA*) respectively.

To isolate the above GA related genes, we constructed *L. perenne* RACE cDNA libraries using plant shoot tissues (sheaths plus blades), following the manufacturer's recommendations (SMART™ RACE cDNA Amplification Kit, Clontech, Norrie Biotech, Auckland, NZ). Specific primers (Supplementary Table 1) for the amplification of 3′- and 5′-fragments were designed from the conserved regions of gene sequences from wheat, barley and rice (QD118250, AB189152, and AB054083 for Lp*GA3ox*; AJ242531, AF460219, and AB262980 for Lp*DELLA*). The 5′- and 3′- fragments of candidate genes were amplified using the Advantage 2 PCR Kit (Clontech, Norrie Biotech, Auckland, NZ). PCR products were cloned (TOPO TA Cloning kit, Invitrogen NZ Ltd, Auckland, NZ) and sequenced at the Allan Wilson DNA Centre, Massey University, Palmerston North, NZ. Full length cDNA sequences were amplified using primers designed from the termini of the target cDNA sequences and cloned and sequenced as described above.

Bioinformatic analyses of the sequences were performed with the software programmes BlastP (http://www.ncbi.nlm.nih.gov/) and ClustalO (http://www.ebi.ac.uk/). The secondary protein structure was determined using a PROSITE programme of Expasy (http://www.expasy.ch/prosite).

### Analysis of gene expression

#### RNA isolation and cDNA synthesis

Total RNA was isolated from plant material using TRIzol Reagent (Invitrogen NZ Ltd., Auckland, NZ) and treated with RNase-free DNase I (Roche NZ Ltd., Auckland, NZ) to remove residual genomic DNA. DNase treated RNA was subsequently purified using the RNeasy Plant Mini Spin Kit (Qiagen, Biostrategy Ltd., Auckland, NZ) to remove enzymes, salts and degraded DNA fragments. RNA quality and integrity was checked by 1% agarose gel electrophoresis, and the absence of genomic DNA confirmed by PCR prior to reverse transcription. RNA was reverse transcribed and converted into cDNA using the SuperScript®VILO™ cDNA Synthesis Kit (Invitrogen NZ Ltd., Auckland, NZ) following the manufacturer's instructions. Synthesized cDNAs were then diluted 50-fold; and 5 μl diluted cDNA was used for subsequent qPCR analysis in a total PCR reaction volume of 20 μl.

#### Transcript profiling by qRT-PCR

Transcript profiling of *L. perenne* genes encoding the fructosyltransferases (Lp*1-SST* (sucrose: sucrose 1-fructosyltransferase, Acc#AY245431; Chalmers et al., 2003) and Lp*6-SFT* (sucrose: fructan 6-fructosyltransferase, Acc#AB186920; Lasseur et al., 2011), a fructan exohydrolase (Lp*1-FEH*, Acc#AY693396), invertases including a vacuolar invertase (Lp*VacInv*, Acc#AY082350), cell wall invertase (Lp*CWInv*, Acc#DQ073969) and cytosolic invertase (Lp*CytInv*, Acc#AM489692), gibberellin synthesizing genes [Lp*GA3ox* (Lp*GA*_3_*-oxidase*, Acc# KP954695) and Lp*GA20ox* (Lp*GA*_20_*-oxidase*, Acc#DQ071620)], and gibberellin inactivating genes [Lp*GA2ox* (Lp*GA*_2_*-oxidase*, Acc#EF687858) and Lp*DELLA*, Acc# KP954694] were quantified by qPCR using a 96-well LightCycler® 480 II system (Roche Diagnostics NZ Ltd., Auckland, NZ). qPCRs were assayed using LightCycler® 480 SYBR Green I Master mix following the manufacturers' protocols (Roche Diagnostics NZ Ltd., Auckland, NZ). For PCR amplification, after an initial 5 min at 95°C, a total of 45 cycles of 10 s at 95°C; 10 s at 60°C; and 10 s at 72°C were performed. The primers for qPCR were designed based either on sequences in the NCBI Genbank (http://www.ncbi.nlm.nih.gov/) or on the isolated genes from our *L. perenne* RACE cDNA libraries (Supplementary Table 1). Three technical replicates were analyzed for each time point and tissue.

Attempts to normalize the transcript data against the means of two *L. perenne* housekeeping genes [elongation factor (Lp*EF1*α) and ubiquitin (Lp*UBQ*)] previously reported to be stably expressed (Martin et al., 2008; Lee et al., 2010; Huang et al., 2014) were unsuccessful as neither of the reference genes were found to be stable in our experimental conditions (see Results). Consequently, an mRNA fragment encoding enhanced Green Fluorescent Protein (eGFP) was artificially synthesized and used as an external reference gene as described previously (Liu and Slininger, 2007; Ellefsen et al., 2008; Rehrig et al., 2011). In the present study, 2 pg eGFP RNA were spiked into 1 μg of sample RNA prior to cDNA synthesis. The gene transcript profiles were normalized against e*GFP* transcripts, and the results are presented as the ratio of target gene copies to e*GFP* copies. Copy numbers were calibrated against the corresponding standard plasmid DNA.

#### Statistical analysis

**Figure 2:**
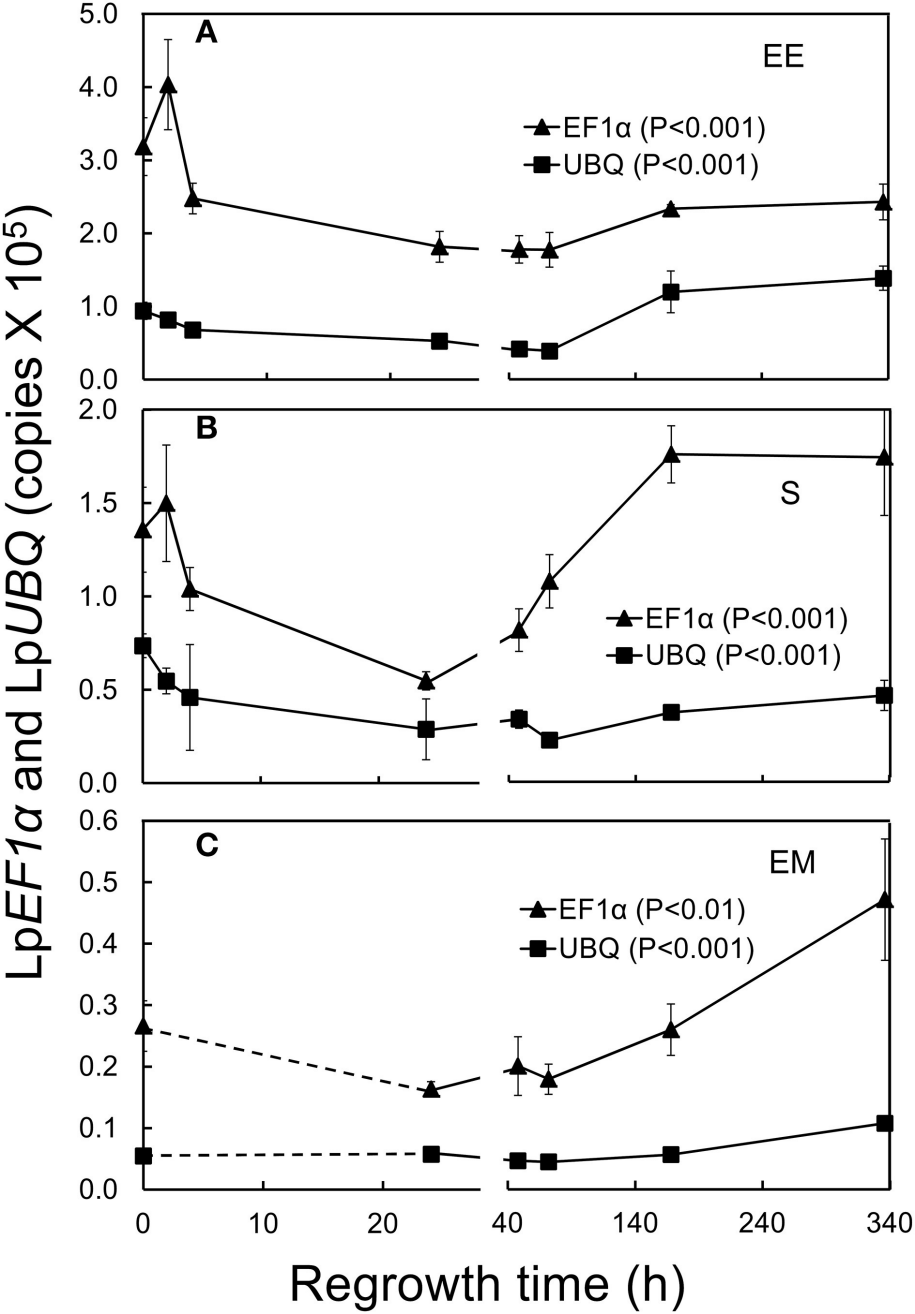
**Changes in elongation factor (Lp***EF1***α; triangles) and ubiquitin (Lp***UBQ***; squares) gene expression in ***L. perenne*** enclosed elongating leaf tissues (EE; A), sheaths (S; B), and emerged leaf tissues (EM; C) after cutting and at subsequent regrowth intervals**. Bars indicate standard errors. For details of Tukey HSD tests see Table 1.

**Figure 3:**
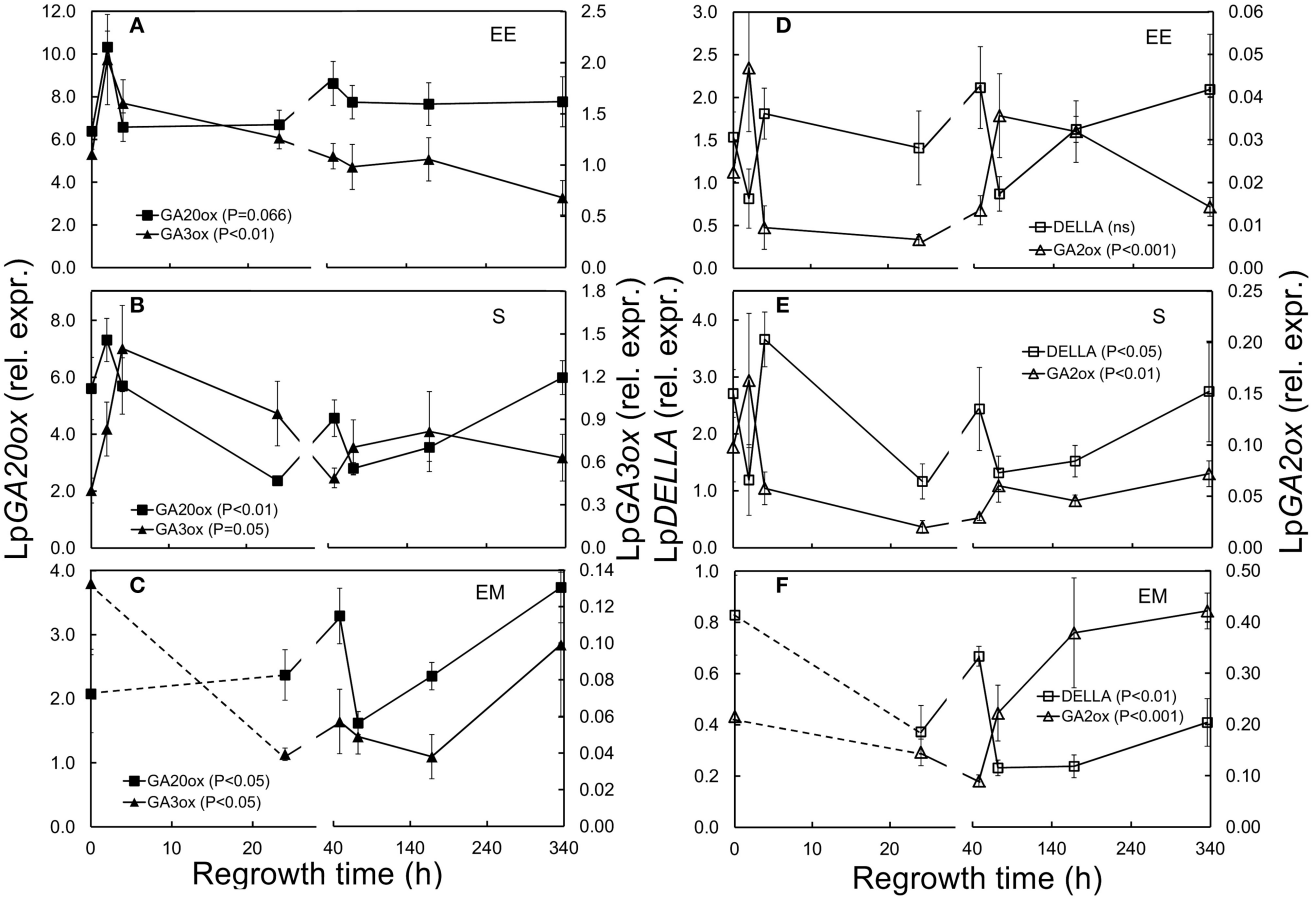
**Expression of GA related genes in ***L. perenne*** enclosed elongating leaf tissues (EE; A,D), sheaths (S; B,E), and emerged leaf tissues (EM; C,F) after cutting and at subsequent regrowth intervals**. Panels on the left show the relative expression of the GA synthesizing Lp*GA3ox* (filled triangles) and Lp*GA20ox* (filled squares). Panels on the right show the relative expression of the GA inactivating Lp*GA2ox* (open triangles) and the GA regulator Lp*DELLA* (open squares). Gene transcript levels were normalized relative to a spiked external reference gene e*GFP* and values presented here have been enlarged 100-fold.

**Figure 4:**
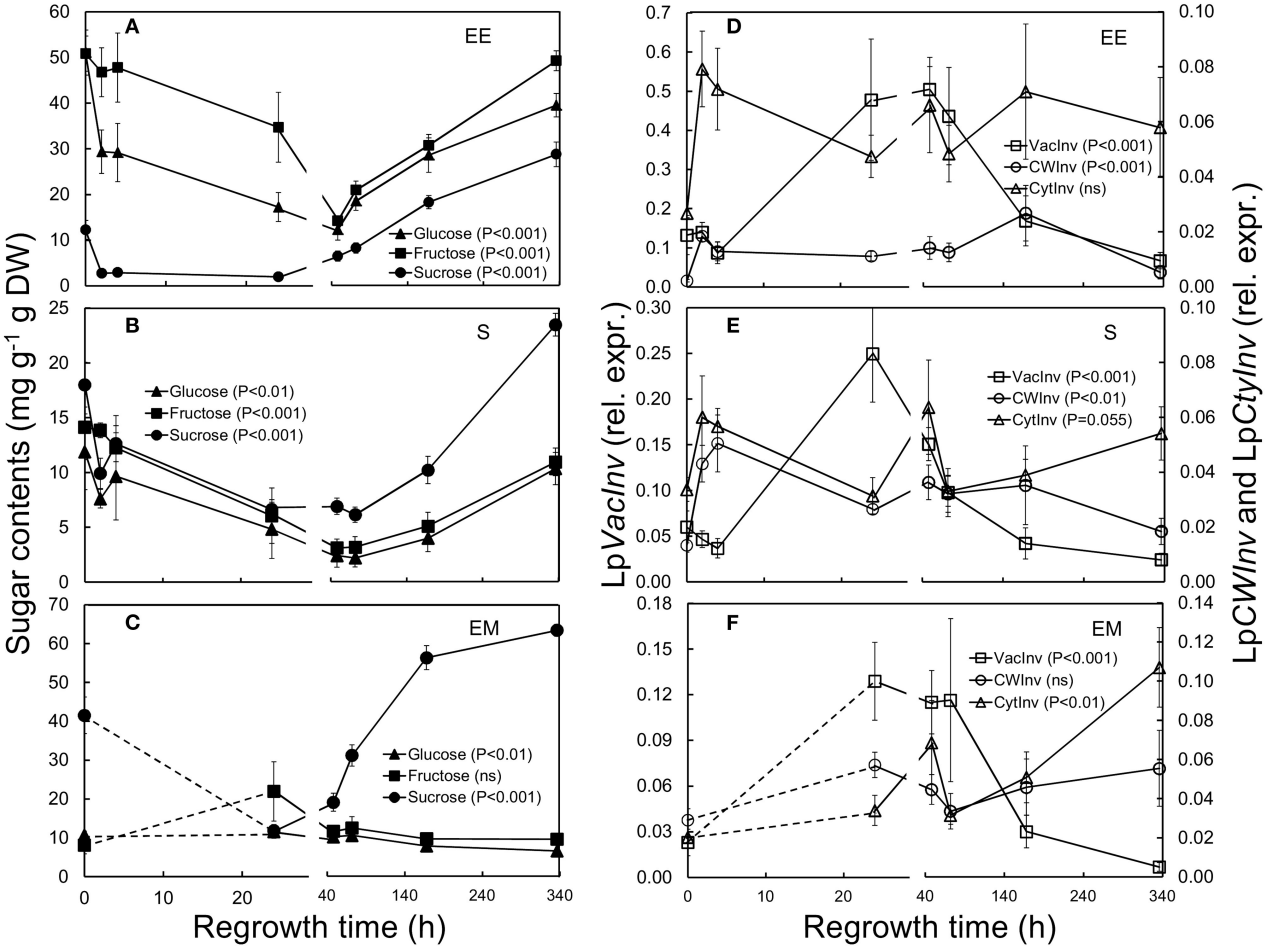
**Sugar concentrations (left panels) and relative expression of invertases (right panels) in ***L. perenne*** enclosed elongating leaf tissues (EE; A,D), sheaths (S; B,E) and emerged leaf tissues (EM; C,F) after cutting and at subsequent regrowth intervals**. Panels on the left show the concentrations of glucose (filled triangles), fructose (filled squares) and sucrose (filled cycles). Panels on the right show the relative expression of Lp*VacInv* (open squares), Lp*CWInv* (open circles), and Lp*CytInv* (open triangles). Gene transcript levels were normalized relative to a spiked external reference gene e*GFP*.

**Figure 5:**
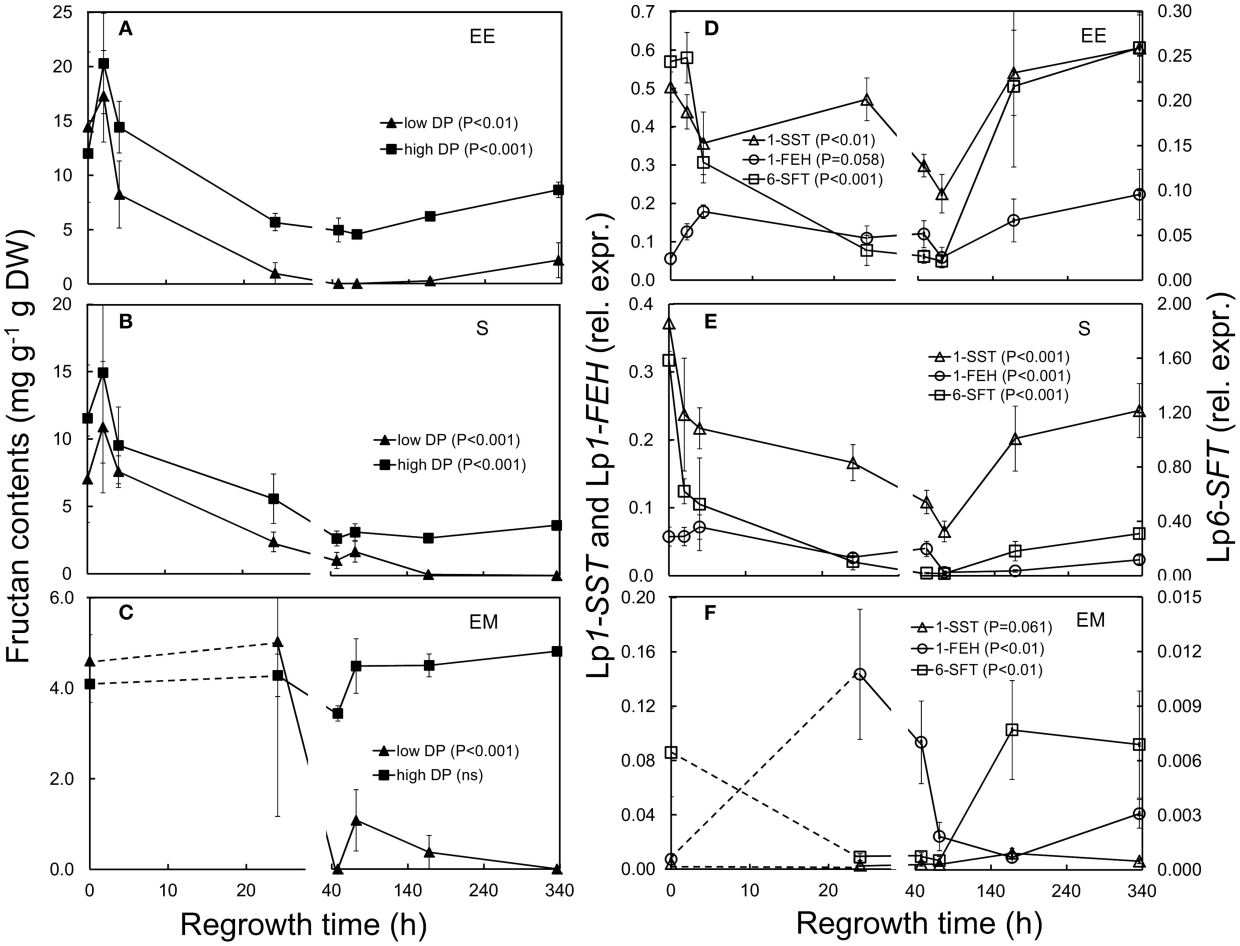
**Fructan concentrations (left panels) and relative expression of fructan-regulating genes (right panels) in ***L. perenne*** enclosed elongating leaf tissues (EE; A,D), sheaths (S; B,E), and emerged leaf tissues (EM; C,F) after defoliation and at subsequent regrowth intervals**. Panels on the left show the concentration of low DP fructans (filled triangles) and high DP fructans (filled squares). Panels on the right show the relative expression of Lp*1-SST* (open triangles), Lp*6-SFT* (open squares) and Lp*1-FEH* (open circles). Gene transcript levels were normalized relative to a spiked external reference gene e*GFP*.

The experiment comprised five biological replicates and eight time points (one pre-defoliation and seven post-defoliation), i.e., a total of 40 plants. Three tissues were analyzed at each time point to give a total of 120 samples. All analyses were conducted by One-way ANOVA to exploit significant differences in individual tissues between the regrowth time points, using Minitab statistical software version 16. We used Box-Cox transformation to homogenize the error variances and, where appropriate, a Tukey's honestly significant different (HSD) mean-separation test was used to help interpret significant effects. We report here untransformed means as a measure of data dispersion in response to treatment effects with the regrowth times and also calculated standard errors (SE) using Minitab (v.16.22), which are shown as bars in Figures 2–**5**. The results of the Tukey's HSD tests are presented in Table 1.

**Table 1:** **Summary of ANOVA results and Tukey honestly significant different tests (different letters represent significant differences)**.

**Tissue**	**Time (h)**	**Low DP fructans**	**High DP fructans**	**Glucose**	**Fructose**	**Sucrose**	***Lp CytInv***	***Lp VacInv***	***Lp CWInv***	***Lp 1-SST***	***Lp 6-SFT***	***Lp 1-FEH***	***Lp GA3ox***	***Lp GA20ox***	***Lp GA2ox***	***Lp DELLA***	***Lp EFα***	***Lp UBQ***
EE	P	<0.01	<0.001	<0.001	<0.001	<0.002	NS^*^	<0.001	<0.001	<0.01	<0.001	0.058	<0.01	0.066	<0.001	NS	<0.001	<0.001
	*F*_(7, 32)_	2.94	14.48	13.73	10.25	42.45		4.75	6.63	4.06	9.46		3.53		6.79		7.85	6.58
	0	ABC	AB	A	A	BC		ABC	C	A	A		AB		AB		AB	A
	2	A	A	BC	A	DE		ABC	A	AB	A		A		A		AB	AB
	4	AB	A	BC	A	DE		BC	AB	AB	AB		A		B		ABC	AB
	24	CD	CD	CD	AB	E		ABC	AB	AB	BC		AB		B		C	BC
	48	D	D	D	C	CD		A	AB	AB	BC		AB		AB		BC	BC
	72	D	D	CD	BC	C		ABC	AB	B	C		AB		A		AB	C
	168	CD	ABC	ABC	ABC	B		ABC	A	A	A		AB		A		A	BC
	336	BCD	ABC	ABC	A	A		C	BC	A	A		B		AB		A	AB
S	P	<0.001	<0.001	<0.01	<0.001	<0.001	= 0.055	<0.001	<0.01	<0.001	<0.001	<0.001	= 0.05	<0.001	<0.01	<0.05	<0.001	<0.001
	*F*_(7, 32)_	5.01	7.51	4.18	6.58	22		9.75	4.32	6.95	11.33	7.1	2.27	7.76	4.72	2.91	6.67	8.41
	0	AB	A	A	A	AB		BC	C	A	A	A	B	ABC	AB	AB	A	ABC
	2	A	A	AB	A	CD		C	AB	AB	A	A	AB	A	A	B	A	ABC
	4	A	A	AB	A	BC		C	A	AB	AB	A	A	AB	ABC	A	AB	ABCD
	24	AB	AB	AB	AB	D		A	ABC	AB	BCD	ABC	AB	D	C	AB	B	CD
	48	ABC	B	B	B	D		AB	AB	BC	CD	ABC	AB	ABC	BC	AB	B	D
	72	ABC	B	B	B	D		BC	ABC	C	D	C	AB	CD	ABC	AB	B	D
	168	BCD	B	AB	AB	CD		C	ABC	AB	ABC	BC	AB	BCD	ABC	AB	AB	AB
	336	C	AB	A	A	A		C	BC	AB	AB	ABC	AB	AB	A	AB	AB	A
EM	P	<0.001	NS	<0.01	NS	<0.001	<0.01	<0.001	NS	0.061	<0.01	<0.01	<0.05	<0.05	<0.001	<0.01	<0.01	<0.001
	*F*_(5, 23)_	3.61		3.95		48.82	5.99	8.75			5.07	4.99	2.74	3.44	7.88	5.71	4.42	6.87
	0	A		A		B	C	AB			A	B	A	AB	ABC	A	AB	B
	24	AB		A		C	ABC	A			AB	A	AB	AB	BC	BC	B	B
	48	B		AB		C	AB	A			AB	AB	AB	AB	C	AB	B	B
	72	AB		AB		B	BC	A			B	B	AB	B	ABC	C	B	B
	168	B		AB		A	ABC	AB			A	B	B	AB	AB	C	AB	B
	336	B		B		A	A	B			A	AB	AB	A	A	ABC	A	A

* *NS, no significant difference*.

The regression analysis was conducted using Minitab (v.16.22) Fitted-Line-Plot.

## Results

### Sequence analysis of GA-related genes

The putative Lp*GA*_3_*-oxidase* (Lp*GA3ox*) mRNA contains an open reading frame (ORF) of 359 amino acids (Supplementary Figure 1), and the deduced amino acid sequence suggests that it belongs to a family of 2-oxoglutarate-dependent dioxygenases which contain conserved sequences, including two histidine residues and an aspartic acid at the cofactor binding sites. The *L. perenne* amino acid sequence shows greatest similarity to GA_3_-oxidases from barley, *Hordeum vulgare* (83%; Spielmeyer et al., 2004), wheat, *T. aestivum* (85%; Appleford et al., 2006), and rice, *Oryza sativa* (77%; Itoh et al., 2001).

Lp*DELLA* encodes a putative GRAS-type transcriptional regulator. The sequence contains an ORF of 612 amino acids which includes the DELLA and TVHYNP motifs at the N-terminal end and a SAW motif at the C-terminal end (Supplementary Figure 2). Alignment of the LpDELLA amino acid sequence with DELLAs from rice (80%; Itoh et al., 2002), wheat (85%; Peng et al., 1999), and barley (84%; Chandler et al., 2002) shows sequence similarity is highly conserved at the C terminal end, while considerable sequence variation occurs at the N-terminus.

### Instability of expression of the “house-keeping” genes Lp*EF*1α and Lp*UBQ*

The expression of Lp*EF1*α and Lp*UBQ* has been shown previously to be relatively stable across a variety of experimental conditions in *L perenne* (Martin et al., 2008; Lee et al., 2010; Huang et al., 2014). Consequently, these genes are often used to “normalize” the expression of target genes in *L. perenne*, i.e., to correct mainly for inefficiencies in cDNA synthesis caused by reverse transcriptase inhibitors possibly present in the sample matrix. Our analysis of the transcript levels of these two genes clearly shows that the expression of Lp*EF1*α and, to a lesser extent, Lp*UBQ* was significantly affected by defoliation resulting in a strong decline in the expression of both of these genes at the early stages of regrowth (Figure 2; Table 1). We therefore normalized the transcript levels of our target genes below against an external control mRNA (eGFP) which was spiked into our quantified RNA samples essentially following the rationale discussed by Rehrig et al. (2011).

Please note that, to make immediate changes (0–24 h post-defoliation) in transcript levels and carbohydrate concentrations better visible, we have split the x-axes in Figures 2–**5** into two parts representing 0–24 h and 48–336 h post-defoliation, respectively. Please also note here and in the following that the first two time points (2 and 4 h) after defoliation could not be analyzed in emerged leaf tissues due to a lack of material.

### Transcriptional regulation of GA related genes following defoliation

Transcript levels of Lp*GA3ox* increased immediately after defoliation in sheaths (Figure 3B; Table 1). In emerged leaf tissues Lp*GA3ox* was not significantly different compared to pre-defoliation levels except 1 week (168 h) after defoliation (Figure 3C; Table 1).

Transcript levels of both Lp*GA20ox* and Lp*GA2ox* were significantly lower in sheaths 24 h after defoliation (0.4-fold Lp*GA20ox* and 0.2-fold Lp*GA2ox*, Table 1). Neither Lp*GA20ox* nor Lp*GA2ox* expression was significantly affected in enclosed elongating and emerged leaf tissues by defoliation (Figures 3C,F).

Lp*DELLA* transcript levels were not significantly affected in enclosed elongating leaf tissues, while in sheaths Lp*DELLA* levels were significantly higher 4 h compared to 2 h after defoliation (Figure 3E; Table 1). In emerged leaf tissues, Lp*DELLA* expression was significantly lower at 24, 72, and 168 h after defoliation (Figure 3F; Table 1).

### Mono- and disaccharide concentrations and expression of invertase genes following defoliation

Overall, sucrose concentrations were lower compared to glucose and fructose in enclosed elongating leaf tissues, while they were higher compared to these monomeric sugars in both mature sheaths and emerged leaf tissues, with very high levels in the latter tissue (Figures 4A–C). Compared to sugar concentrations prior to defoliation, sucrose concentrations dropped sharply in the first 2 h after defoliation (*P* < 0.01) in enclosed elongating leaf tissues and sheaths (Figures 4A,B; Table 1) reaching lowest levels after 24 h. These low sucrose concentrations were also seen in emerged leaf tissues 24 and 48 h after defoliation (Figure 4C). Glucose concentrations were also significantly lower already 2 h after defoliation, but only in enclosed elongating leaf tissues (Figure 4A), while they dropped only slightly in sheaths (Figure 4B). Lowest glucose levels were observed in both these tissues 48 h after defoliation. The concentration of fructose in enclosed elongating leaf tissues and sheaths remained at pre-defoliation levels in the first 24 h and dropped to their lowest levels 48 h after defoliation (Figures 4A,B), while they were not significantly affected in emerged leaf tissues (Figure 4C).

The expression of the vacuolar invertase gene (Lp*VacInv*) increased significantly in sheaths 24 h after defoliation (Figures 4D–F; Table 1). The expression of Lp*VacInv* was logarithmically and negatively linked to sucrose concentrations for all tissues tested across the regrowth period [sucrose = −6.53 log(Lp*VacInv*) + 0.3921, *R*^2^ = 0.316, *P* < 0.001; Supplementary Figure 3A].

An increase in Lp*CWInv* expression was also observed and this occurred already 2 h after cutting in enclosed elongating leaf tissues and sheaths (*P* < 0.01, Figures 4D,E; Table 1), while no difference in emerged leaf tissues was detected. In contrast, increased expression of the cytosolic invertase (Lp*CytInv*) was also detected, but only in emerged leaf tissues 48 h following defoliation (*P* < 0.01, Figure 4F; Table 1).

Interestingly, in this study a significant negative association was seen between sucrose concentrations and the expression of the GA-activating Lp*GA3ox* [sucrose = −7.806 log(Lp*GA3ox*) −24.931, *R*^2^ = 0.408, *P* < 0.001; Supplementary Figure 3B]; and a positive association between sucrose and the GA-inactivating Lp*GA2ox* [sucrose = −983,886(Lp*GA2ox*)^2^ + 14,371(Lp*GA2ox*) + 6.5402, *R*^2^ = 0.574, *P* < 0.001] was observed for all tested tissues (Supplementary Figure 3C).

### Water soluble carbohydrate concentrations and transcriptional expression of fructan related genes following defoliation

WSC concentrations were investigated by determining LMW [mixture of low degree of polymerisation (DP) fructans, sucrose, glucose, and fructose] and HMW WSC (high DP fructans) fractions using anthrone as the detecting reagent. To deduce the concentration of low DP fructans, the sum of glucose, fructose, and sucrose (quantified by enzymatic analysis, Rasmussen et al., 2008) was subtracted from the total LMW WSC concentration. Overall low DP fructans were lower compared to high DP fructans in all tissues analyzed (Figures 5A–C).

In enclosed elongating leaf tissues concentrations of low and high DP fructans decreased significantly (low DP fructans 48–72 h; high DP fructans 24–72 h) after defoliation, and reached pre-defoliation levels after a week (168 h, Figure 5A; Table 1). In sheaths, high DP fructans were significantly lower 48–168 h post-defoliation (Figure 5B; Table 1). In emerged leaf tissues concentrations of low DP fructans also decreased significantly 48 h after defoliation (Figure 5C; Table 1) and remained low during the course of the experiment. Concentrations of high DP fructans were not significantly affected by defoliation in this tissue.

In association with the changes in fructans following defoliation, transcript levels of fructan synthesizing Lp*1-SST* and Lp*6-SFT*, were significantly reduced in enclosed elongating leaf tissues (*P* < 0.01, Figure 5D; Table 1) and sheaths (*P* < 0.001, Figure 5E; Table 1). In both tissues expression of Lp*1-SST* and Lp*6-SFT* was lowest 72 h after cutting, then generally increased to the levels seen prior to defoliation after 1 week. Significantly decreased Lp*6-SFT* transcript levels also occurred in emerged leaf tissues during the first 72 h after cutting (*P* < 0.01), but there was no significant change observed for Lp*1-SST* in this tissue. As expected, a significant positive association between high DP fructan concentrations and Lp*6-SFT* gene expression was observed in enclosed elongating leaf tissues and sheaths [in enclosed elongating leaf tissues: High DP fructans = 15.663(Lp*6-SFT*)^0.2544^, *R*^2^ = 0.4537, *P* < 0.001, Supplementary Figure 3D; in sheaths: High DP fructans = −3.2407(Lp*6-SFT*)^2^ + 14.596(Lp*6-SFT*) + 2.7459, *R*^2^ = 0.3939, *P* < 0.001, Supplementary Figure 3E].

In this study, the expression of Lp*1-FEH* was significantly higher in emerged leaf tissues 24 h following defoliation (Figure 5F). However, compared to emerged leaf tissues at T0, which consist mainly of mature blade tissue, the majority of this leaf category at 24 h post-defoliation had only recently emerged, i.e., represents very young tissues and might not be directly comparable to T0 of the same category. The increase in Lp*1-FEH* gene expression in enclosed elongating leaf tissues was marginally significant (*P* = 0.058; Figure 5D; Table 1). There was no change detected in sheaths in the earlier stages, but expression of Lp*1-FEH* was significantly reduced 72–168 h post-defoliation (Figure 5E).

## Discussion

In a recent review (Parsons et al., 2011) we highlighted that although there is an urgent need to increase dry matter production of forage grasses, while at the same time limiting adverse environmental impacts caused by excessive use of N fertilization, there are only a few examples of forage breeding strategies targeted at the very processes (photosynthesis, respiration, N uptake and utilization) acting at the core of plant growth. In a previous study (Parsons et al., 2013a) we used exogenous application of GA_3_ to successfully stimulate *L. perenne* growth without the need for additional resources (N), although this was dependant on seasonal development. We postulated that GA_3_ might act at the center of vegetative growth regulation in this important forage grass particularly during regrowth after defoliation. To test this hypothesis further, we analyzed in the present study the expression of GA metabolic pathway and regulatory genes, and their possible relationship to carbohydrate, especially fructan metabolism.

### Transcriptional regulation of GA-biosynthesis and degradation genes in response to defoliation

The plant hormone gibberellin (GA) acts as a key mediator between environmental cues and plant morphology in a variety of developmental processes, including stem and root elongation, seed germination, and floral development (see reviews Yamaguchi, 2008; Colebrook et al., 2014). Numerous studies, particularly in *Arabidopsis*, rice, barley, and wheat demonstrated that the biologically active GA_1_, GA_3_ and GA_4_ are synthesized by the 2-oxoglutarate-dependent dioxygenases GA_20_-oxidase and GA_3_-oxidase, but inactivated by the degrading enzyme GA_2_-oxidase (see reviews Yamaguchi, 2008; Harberd et al., 2009; Hedden and Thomas, 2012). GA activity is also regulated by transduction regulators of growth, the repressing DELLA protein (Peng et al., 1999) and GA receptors such as GID1 (gibberellin insensitive dwarf; Ueguchi-Tanaka et al., 2005; Chandler et al., 2008). To date, however, only an Lp*GA*_2_*- oxidase* (EF687858; MacMillan et al., 2005) and an Lp*GA*_20_*- oxidase* (DQ071620, King et al., 2008) have been isolated from *L. perenne*.

In the present study, we isolated genes encoding a putative Lp*GA*_3_*-oxidase*, which catalyzes the latter steps of GA activation, and a putative Lp*DELLA* which is a growth repressing regulator of the GA signaling pathway. Our study showed that there was a significant up-regulation of Lp*GA3ox* in sheaths following defoliation. Expression of both Lp*GA20ox* and Lp*GA2ox* was significantly down-regulated in sheaths 24 h post-defoliation. Reduced levels of Lp*GA2ox* were also observed in enclosed elongating blades, but the reduction was only significant comparing the 4 and 24 h time points to the 2 h post-defoliation time point. The expression of DELLA, a repressor of GA responses, was initially reduced followed by an up-regulation, but this was statistically significant in mature sheaths (when comparing the 4 h with the 2 h time point) and in emerged leaf tissues only. Taken together our results indicate that defoliation of ryegrass does result in a differential expression of GA activating and inactivating enzymes, but due to high between replicate variability of the expression of the tested genes, and therefore lack of statistical significance, our study provides only indicative evidence of defoliation effects on GA metabolism. The expression of GA-related genes in response to environmental triggers has been shown previously to be very rapid and transient (Yamaguchi et al., 1998), and the high variability seen in our study might have been a result of the relative long time (15–20 min per plant) it takes to separate enclosed elongating leaf tissues from surrounding sheaths.

It needs to be noted here that GA_3_ has never been demonstrated to be present in *L. perenne*, while GA_1_, another active gibberellin, has been shown to be decreased after defoliation (Morvan-Bertrand et al., 2001). On the other hand, exogenous application of GA_3_ has been shown to induce stem elongation (MacMillan et al., 2005) and to increase dry matter production (Parsons et al., 2013a), and it is possible that levels of GA_3_ are too low to be detected by common analytical methods or GA_3_ might be located in very small tissue complexes, such as the basal leaf meristems, only. Another possibility might be that exogenously applied GA_3_ is subsequently converted *in planta* to other growth-stimulating gibberellins, but clearly more research is needed.

### Carbohydrate metabolism and its transcriptional regulation in response to defoliation

Defoliation is a major event in terms of *L. perenne* growth in an agricultural context (Morvan et al., 1997; Pilon-Smits et al., 1999; Wei and Chatterton, 2001; Lee et al., 2011), because the removed photosynthetic tissue needs to be compensated for as soon as possible in order to restore plant maintenance and sugar storage to pre-defoliation levels. This can only be achieved by elongation and eventual emergence of the enclosed immature blades, which requires the supply of carbohydrates to this heterotrophic tissue. Our results demonstrate that glucose and sucrose are rapidly depleted in immature blades, already 2 h after defoliation sucrose levels decreased to almost zero in this tissue. Insufficient new photosynthetically active tissue has emerged at this time and, in the absence of *de novo* synthesized sugars, carbohydrates have to be mobilized from their storage forms in the remaining tissues.

In *L. perenne* the two major storage carbohydrates are sucrose and fructans (Pollock and Cairns, 1991; Pavis et al., 2001a,b). To provide glucose, sucrose needs to be cleaved by invertases into glucose and fructose and we show here that the expression of all three forms of invertases, i.e., vacuolar, cytosolic and cell wall invertases are up-regulated after defoliation. The first invertase gene to be significantly up-regulated after 2 h was Lp*CWInv* in both enclosed immature blades and mature sheaths, and we suggest that the immediate drop in sucrose in these two tissues is caused by an increased activity of invertases located in the cell walls (Sherson et al., 2003; Lammens et al., 2008; Proels and Hückelhoven, 2014). A second invertase gene, i.e., Lp*VacInv*, was up-regulated much later (24 h post-defoliation) with expression particularly high in newly emerged blades, and we hypothesize that this type of invertase serves to hydrolyse *de novo* synthesized sucrose. Both, the hydrolysis of sucrose and of fructans also releases fructose, and concentrations of this monomeric sugar remained relatively constant in the first 24 h after defoliation and only decreased at later time points possibly indicating that fructose cannot be used directly for growth and that isomerases transforming it into glucose are relatively slowly up-regulated.

The second major storage carbohydrate in *L. perenne* are fructans (Pollock and Cairns, 1991; Smouter and Simpson, 1991; Morvan-Bertrand et al., 2001) and we found high levels of fructans in enclosed elongating blades and in mature sheaths prior to defoliation, indicating that these tissues act as strong sinks for photosynthates (Schnyder et al., 1988; Spollen and Nelson, 1988; Allard and Nelson, 1991). Following defoliation we saw a reduction in both low and high DP fructans, but this took place much later than the reduction in sucrose, between 24 (enclosed blades) and 48 h (mature sheaths) post-defoliation.

Degradation of fructans after defoliation in both enclosed and emerged blades of *L*. *perenne* has been reported to be associated with an increased enzymatic activity of fructan exohydrolase (FEH) which catalyzes fructan breakdown (Morvan et al., 1997; Chalmers et al., 2005; Lothier et al., 2007, 2014). Lp*1-FEH* gene expression was also reported to be negatively correlated with fructan accumulation and the expression of fructosyltransferases in both mature blades and sheaths (Morvan et al., 1997; Rasmussen et al., 2014). In the present study, we observed 24 h post-defoliation a higher Lp*1-FEH* gene expression compared to T0 in the emerged leaf tissue category. However, this tissue consists mainly of very young tissue which had at the earlier time points been enclosed elongating leaf tissue and comparing the levels of 1-FEH transcripts with those found in enclosed emerging tissue 2 and 4 h post-defoliation, levels of 1-FEH were actually lower.

Strongly declined transcript levels of genes encoding enzymes responsible for synthesizing reserve carbohydrates (Lp*1-SST* and Lp*6-SFT*) were observed following defoliation, which is consistent with previous observations on fructan accumulation in perennial ryegrass (Morvan et al., 1997; Lidgett et al., 2002; Hisano et al., 2008). As anticipated, we found that the relative transcript levels of fructan biosynthesizing genes, particularly Lp*6-SFT*, correlated positively with the accumulation of high DP fructans in both enclosed elongating blades and mature sheaths.

Taken together, our results suggest that after defoliation in mature sheaths and enclosed blades the immediate glucose demand of elongating blades is met by hydrolysis of sucrose first through cell wall and subsequently vacuolar invertases. Degradation of any pre-formed fructans and inhibition of *de novo* fructan synthesis are more long term processes occurring during 24–72 h post-defoliation. A full recovery of sugar and sugar metabolism related gene transcript levels to pre-defoliation levels is achieved between 1 and 2 weeks post-defoliation.

### Insights into grass growth—sugar signaling and hormone interaction

Low nutrient levels including low sucrose and glucose concentrations have been shown to activate GA biosynthesis and repress its degradation in several plant systems including cereal seed germination (Bewley and Black, 1985; Gibson, 2004; Hong et al., 2012; Colebrook et al., 2014). Increased levels of active GAs in turn induce a plethora of macromolecule degrading enzymes such as proteases, lipases and α-amylases (in starch accumulating plants/tissues). In our study, defoliation of *L. perenne* also resulted in a dramatic and very rapid depletion of glucose and sucrose pools. This depletion might also have caused an increase in genes coding for GA-activating enzymes, but because this increase was statistically significant only for Lp*GA3ox* in mature sheaths, our results are not conclusive. However, a strong negative association between sucrose and transcript levels of Lp*GA3ox* as well as a strong positive association between sucrose and levels of Lp*GA2ox* might be seen as additional evidence for signaling between low sucrose levels and GA-activation.

Induction of GA biosynthesis as well as exogenous supply of GA_3_ to barley and rice seeds were shown to induce the expression of more than 1000 genes encoding e.g., hydrolases, proteases, and lipases (Chen and An, 2006; Tsuji et al., 2006; Hong et al., 2012) and also sucrolytic activity (see review Roitsch and González, 2004). In our study we saw a strong and very rapid increase in Lp*CWInv* (2 h post-defoliation) in both enclosed elongating blades and mature sheaths, from our data it is unlikely that this increase was induced by active GA as induction of Lp*GA3ox* and Lp*GA20ox* was seen at the same or later time points. Although it is possible that, at least for Lp*GA3ox*, we might have missed the time of highest expression, which might have been earlier than 2 h post-defoliation, our results do not allow a clear statement regarding these events. However, we did see a strong increase in Lp*VacInv* expression 24 h post-defoliation which might have prevented sucrose build-up in sheaths and emerged mature blades at the early stages of re-growth (after formation of new, photosynthetically active tissue). We saw a strong down-regulation of fructan biosynthesizing genes following defoliation, and it would be interesting to test if the expression of these genes is repressed by GAs. Future studies should therefore aim to isolate promoter regions of carbohydrate related genes from *L. perenne* and perform promoter binding studies (see Hong et al., 2012).

Summarizing, we propose that our study indicates that sugar depletion following defoliation might affect GA-biosynthesis, but that our results are not always conclusive and additional experiments are needed to unequivocally show a link between sugars and GA in perennial ryegrass during re-growth after defoliation.

## Concluding remarks

Our results here and those of a previous study (Parsons et al., 2013a) provide indicative evidence that gibberellins might play a role in *L. perenne* re-growth after defoliation, but that further studies are needed to unambiguously demonstrate a link between defoliation, sugar depletion and GA activation. Increasing ryegrass vegetative (dry matter) production is at the center of efforts to increase pasture based animal production. Because a strong link between vegetative production and gibberellins has been shown in cereals, we propose that gibberellin signaling in perennial ryegrass should be studied in more detail to possibly improve our chances of achieving this goal.

## Author contributions

All authors contributed to the research and manuscript equally; and read and approved the final version of the manuscript. All authors agree to be accountable for all aspects of the work.

### Conflict of interest statement

The authors declare that the research was conducted in the absence of any commercial or financial relationships that could be construed as a potential conflict of interest.

## Abbreviations

GA: gibberellin
WSC: water soluble carbohydrate
DP: degree of polymerisation
FEH: fructan exohydrolase
SST: sucrose: sucrose fructosyltransferase
SFT: sucrose: fructan fructosyltransferase.
