# Supplementary material for: Does gibberellin biosynthesis play a critical role in the growth of Lolium perenne? Evidence from a transcriptional analysis of gibberellin and carbohydrate metabolic genes after defoliation
Source: Front Plant Sci. 2015 Nov 3;6:944. doi: 10.3389/fpls.2015.00944 (PMC4630572; doi:10.3389/fpls.2015.00944)
Supplement: Supplementary file 1 [file DataSheet1.PDF]

Supplementary Table 1 Primer sequences used in this study for RT-qPCR and 5' and 3' gene fragment amplification of target genes.

Primer name	Target gene	Sequence (5' to 3')	Size (bp)	Reference
CWInv_for	qPCR	CTGCCGACCTAGCAGAGAAG	82	DQ073969
CWInv_rev	Lp*CWInv*	GCTGCACATGAGGACGATAG		
VacInv_for	qPCR	TCTTCCTAGTTGCTTCCATCG	92	AY082350
VacInv_rev	Lp*VacInv*	CGCATACGAGTAGGGCATC		
CytInv_for	qPCR	CTCTGGAACGACGAGGACAC	102	AM489692
CytInv_rev	Lp*CytInv*	TGCCGAGAACTCACACAATG		
1-SST_for	qPCR	AAGTCCTCCGGTGCCTACTC	196	AY245431
1-SST_rev	Lp*1-SST*	GCGATGTTGCCCCAGCT		
6-SFT_for	qPCR	GTTCTCGCTCACCCACAG	186	AB186920
6-SFT_rev	Lp*6-SFT*	GACACGCTCGAAGCTAAGG		
1-FEH-for	qPCR	AAGGTGCCAAACATGTCCTC	239	DQ073968
1-FEH-rev	Lp*1-FEH*	TGCGACGTCATCTGAAGAAC		
GA3ox_for	qPCR	TGTGAGGTGATGGAGGAGTTC	165	This study
GA3ox_rev	Lp*GA3ox*	GTACCAGTTGAGGTGCATGG		
GA20ox_for	qPCR	AGGTGTACGCTCGGTACTGC	136	DQ071620
GA20ox_rev	Lp*GA20ox*	TTGAGCCGCATTATGGATTC		
GA2ox_for	qPCR	GGTGGATCGAGTACCTCCTG	81	EF687858
GA2ox_rev	Lp*GA2ox*	ACGGCAATGTCGAGAACG		
DELLA_for	qPCR	CATCCTCCTCCTCGTCCTC	200	This study
DELLA_rev	Lp*DELLA*	GAGCGGTATCTGCTTGACG		
eGFP_for	qPCR	CATCGAGCTGAAGGGCATC	84	This study
eGFP_rev	e*GFP*	GACGTTGTGGCTGTTGTAGTTG		
EF1α_for	qPCR	GGCTGATTGTGCTGTGCTTA	114	EU168438
EF1α_rev	Lp*EF1α*	CTCACTCCAAGGGTGAAAGC		
UBQ_for	qPCR	AGAAGGAGTCCACCCTCCAC	111	EF470423
UBQ_rev	Lp*UBQ*	TCACCTTCTTGTGCTTGTGC		
GA3ox_3'	Lp*GA3ox*	ATCTCCTCCTTCTTCTCCAAGTGCATGT		This study
GA3ox_5'		AGCACGAAGGTGAAGAAGCCCGAGT		
Della_3'	Lp*DELLA*	CCTAACGAGGAGCCCGAGGTAATC		This study
Della_5'		AGGGAGTCGAACATGGTGGAGTAGTA		

```
LpGA3ox            MPTPSHLSSKDPRYFDFRAARRVPETHAWPGLHDHPVVDGGH-SAGEDAVPVVDMRDPGA      59
Rice_BAB62072      MPTPSHL--KNPLCFDFRAARRVPETHAWPGLDDHPVVDGGG-GGGEDAVPVVDVRAGDA      57
Barley_AAT49061    MPTPSHLS-KDPHYFDFRAARRVPETHAWPGLHDHPVVDGGGAGGGPDAVPVVDMRDPCA      59
Wheat_AAZ94377     MPTPSHLS-KDPRYFDFRAARRVPETHAWPGLHDHPVVDGSGAGGGPDAVPVVDMRDPCA      59
                   *******  *:*  *****************.*******.   ..* *******:*   *

LpGA3ox            AEAVARAAEQWGAFLLQGHGVPHELLARVEARIAGMFALPATEKMRAVRRPGDSCGYGSP      119
Rice_BAB62072      AARVARAAEQWGAFLLVGHGVPAALLSRVEERVARVFSLPASEKMRAVRGPGEPCGYGSP      117
Barley_AAT49061    AEAVALAAQDWGAFLLQGHGVPLELLARVEAAIAGMFALPASEKMRAVRRPGDSCGYGSP      119
Wheat_AAZ94377     AEAVALAAQDWGAFLLEGHGVPLELLAGVEAAIGGMFALPASEKMRAVRRPGDSCGYGSP      119
                   *  ** **::****** *****  **: **  :. :*:***:******* **: ******

LpGA3ox            PISSFFAKSMWSEGYTFSPANLRSDLRRLWPKQGHDYRLFCEVMEEFHGEMRALSDRLME      179
Rice_BAB62072      PISSFFSKLMWSEGYTFSPSSLRSELRRLWPKSGDDYLLFCDVMEEFHKEMRRLADELLR      177
Barley_AAT49061    PISSFFSKCMWSEGYTFSPANLRSDLRKLWPKAGHDYRHFCAVMEEFHREMRVLADKLLE      179
Wheat_AAZ94377     PISSFFSKCMWSEGYTFSPANLRSDLRKLWPKAGHDYRHFCAVMEEFHREMRALADKLLE      179
                   ******:* **********:.***:**:**** *.**  ** ****** *** *:*.*:.

LpGA3ox            LFLAALGLTGEQAAAVEAEHRIAETMTATMHLNWYPKCPDPKRALGLIAHTDSGFFTFVL      239
Rice_BAB62072      LFLRALGLTGEEVAGVEAERRIGERMTATVHLNWYPRCPEPRRALGLIAHTDSGFFTFVL      237
Barley_AAT49061    LFLVALGLTGEQVAAVESEHKIAETMTATMHLNWYPKCPDPKRALGLIAHTDSGFFTFVL      239
Wheat_AAZ94377     LFLVALGLTGEQVAAVESEHKIAETMTATMHLNWYPKCPDPKRALGLIAHTDSGFFTFVL      239
                   *** *******:.*.**:*::*.* ****:******:**:*:*****************

LpGA3ox            -------QLFRHGPDRRVTVPAEARDAFVVNVGDLFQILTNGRFHSVYHRAVVNCDSDRI      292
Rice_BAB62072      QSLVPGLQLFRRGPDRWVAVPAVA-GAFVVNVGDLFHILTNGRFHSVYHRAVVNRDRDRV      296
Barley_AAT49061    QSLVPGLQLFRHGPDRWVTVPAVP-GAMVVNVGDLFHILTNGRFHSVYHRAVVNRDSDRI      298
Wheat_AAZ94377     QSLVPGLQLFRHGPDRWVTVPAVP-GAMVVNVGDLFQILTNGRFHSVYHRAVVNRDSDRI      298
                          ****:**** *:***    *:********:***************** * **:

LpGA3ox            SLGYFLGPPADTKVAPLREA---GGKPAYRAVTWPEYMAVRKKAFTTGASALKMVSASTD      349
Rice_BAB62072      SLGYFLGPPPDAEVAPLPEAVPAGRSPAYRAVTWPEYMAVRKKAFATGGSALKMVSTDAA      356
Barley_AAT49061    SLGYFLGPPAHVKVAPLREALA-GTPAAYRAVTWPEYMGVRKKAFTTGASALKMVAISTD      357
Wheat_AAZ94377     SLGYFLGPPAHVKVAPLREALA-GTPAAYRAVTWPEYMGVRKKAFTTGASALKMVAISTD      357
                   ********* ..:**** **   *   ***********.******:**.******: .:

LpGA3ox            DDDLS--D-LISS----- 359
Rice_BAB62072      AAADE-HDDVAAAADVHA 373
Barley_AAT49061    DAADVLPDVLSS------ 369
Wheat_AAZ94377     NDAANDTDDLISS----- 370
                            * : :
```

Supplementary Figure 1 ClustalO alignment of the deduced LpGA$_3$-oxidase with GA*3*-oxidases from *O. sativa* (Acc. # BAB62072; 77% id.), *H. vulgare* (Acc. # AAT49061; 83% id.), and *T. aestivum* (Acc. # AAZ94377; 85% id.). Identity (*), strongly similar (:), and weakly similar (.).

```
LpDELLA           MEREYQDAGGSSAAAA----GMSKDKMMLSAPPPQEDEDVDELLAALGYKVRSSDMADVA   56
Rice_BAE96289     MKREYQEAGGSSGGGSSADMGSCKDKVM--AGAAGEEEDVDELLAALGYKVRSSDMADVA   58
Barley_AAL66734   MKREYQDGGGSGGGGDE--MGSSRDKMMVSSSEAGEGEEVDELLAALGYKVRASDMADVA   58
Wheat_CAB51555    MKREYQDAGGSGGGGGG--MGSSEDKMMVS-AAAGEGEEVDELLAALGYKVRASDMADVA   57
                  *:****:.***....     * ..**:*       * *:*************:*******

LpDELLA           HKLEQLEMAMGMGAV---PAPDDGFTTHLATETVHYNPTDLSSWVESMLSELNAPPPPLP   113
Rice_BAE96289     QKLEQLEMAMGMGGVSAPGAADDGFVSHLATDTVHYNPSDLSSWVESMLSELNAPLPPIP   118
Barley_AAL66734   QKLEQLEMAMGMGG----PAPDDGFATHLATDTVHYNPTDLSSWVESMLSELNAPPPPLP   114
Wheat_CAB51555    QKLEQLEMAMGMGGVGAGAAPDDSFATHLATDTVHYNPTDLSSWVESMLSELNAPPPPLP   117
                  :************.     * **.*.:****:******:**************** **:*

LpDELLA           PAPASS-------TVT---ADGFFDIPPPSIDSSSTSYALRPIPSPPVD-----LSADSV   158
Rice_BAE96289     PAPPAARHASTSSTVTGGGGSGFFEL-PAAADSSSSTYALRPISLPVVAT--ADPSAADS   175
Barley_AAL66734   PAPPQLNA-STSSTVTG--GGGYFDL-PPSVDSSSSTYALRPIISPP--VAPADLSADS-   167
Wheat_CAB51555    PA-PQLNA-STSSTVTG--SGGYFDL-PPSVDSSSSIYALRPIPSPAGATAPADLSADS-   171
                  **           ***   . *:*:: * : ****: ******  *         ** .

LpDELLA           PRDPKRMRTGGGSTSSSSSSSSSSLG----GCVVEAAPPAAE--ANANAIALPVVVADTQ   212
Rice_BAE96289     ARDTKRMRTGGGSTSSSSSSSSSLGGGASRGSVVEAAPPATQGAAAANAPAVPVVVVDTQ   235
Barley_AAL66734   VRDPKRMRTGGSSTSSSSSSSSSLGGGAARSSVVEAAPPVA---AAAAAPALPVVVVDTQ   224
Wheat_CAB51555    VRDPKRMRTGGSSTSSSSSSSSSLGGGAR-SSVVEAAPPVAA--AANATPALPVVVVDTQ   228
                   ** *******.***********  *    ..*******.:   *   : *:****.***

LpDELLA           EAGIRLVHALLACAEAVQQENFPAAEALVKQIPLLAASQGGAMRKVAAYFGEALARRVFR   272
Rice_BAE96289     EAGIRLVHALLACAEAVQQENFAAAEALVKQIPTLAASQGGAMRKVAAYFGEALARRVYR   295
Barley_AAL66734   EAGIRLVHALLACAEAVQQENLSAAEALVKQIPLLAASQGGAMRKVAAYFGEALARRVFR   284
Wheat_CAB51555    EAGIRLVHALLACAEAVQQENLSAAEALVKQIPLLAASQGGAMRKVAAYFGEALARRVFR   288
                  *********************: ********** ************************:*

LpDELLA           FRPQPDSSHLDAAFADLLHAHFYESCPYLKFAHFTANQAILEAFAGCRRVHVVDFGIKQG   332
Rice_BAE96289     FRPA-DSTLLDAAFADLLHAHFYESCPYLKFAHFTANQAILEAFAGCHRVHVVDFGIKQG   354
Barley_AAL66734   FRPQPDSSLLDAAFADLLHAHFYESCPYLKFAHFTANQAILEAFAGCRRVHVVDFGIKQG   344
Wheat_CAB51555    FRPQPDSSLLDAAFADLLHAHFYESCPYLKFAHFTANQAILEAFAGCRRVHVVDFGIKQG   348
                  ***  **: **************************************:************

LpDELLA           MQWPALLQALALRPGGPPSFRLTGVGPPQPDETDALQQVGWKLAQFAHTIGVDFQYRGLV   392
Rice_BAE96289     MQWPALLQALALRPGGPPSFRLTGVGPPQPDETDALQQVGWKLAQFAHTIRVDFQYRGLV   414
Barley_AAL66734   MQWPALLQALALRPGGPPSFRLTGVGPPQPDETDALQQVGWKLAQFAHTIRVDFQYRGLV   404
Wheat_CAB51555    MQWPALLQALALRPGGPPSFRLTGVGPPQPDETDALQQVGWKLAQFAHTIRVDFQYRGLV   408
                  ************************************************** *********

LpDELLA           AATLADLEPFMLQPEAEDGPNEEPEVIAVNSVFEMHRLLAQPGALEKVLGTVRAVRPRIV   452
Rice_BAE96289     AATLADLEPFMLQPEGEADANEEPEVIAVNSVFELHRLLAQPGALEKVLGTVHAVRPRIV   474
Barley_AAL66734   AATLADLEPFMLQPEGEEDPNEEPEVIAVNSVFEMHRLLAQPGALEKVLGTVRAVRPRIV   464
Wheat_CAB51555    AATLADLEPFMLQPEGEEDPNEEPEVIAVNSVFEMHRLLAQPGALEKVLGTVRAVRPRIV   468
                  ***************.*   **************:****************:********

LpDELLA           TVVEQEANHNTGSFLDRFTESLHYYSTMFDSLEGAG--SGPSEISSGSSAAAAAANAAPG   510
Rice_BAE96289     TVVEQEANHNSGSFLDRFTESLHYYSTMFDSLEGGSSGQAELS--------PPAAGGGGG   526
Barley_AAL66734   TVVEQEANHNSGSFLDRFTESLHYYSTMFDSLEGGSS-GGPSEVSSGGA----APAAAAG   519
```

```
Wheat_CAB51555      TVVEQEANHNSGTFLDRFTESLHYYSTMFDSLEGGSSGGGPSEVSSGAA----AAPAAAG    524
                    **********:*:*********************..   .  .          *  .. *

LpDELLA             TDQVMSEVYLGRQICNVVACEGAERTERHETLGQWRGRLGHAGFETVHLGSNAYKQASTL    570
Rice_BAE96289       TDQVMSEVYLGRQICNVVACEGAERTERHETLGQWRNRLGRAGFEPVHLGSNAYKQASTL    586
Barley_AAL66734     TDQVMSEVYLGRQICNVVACEGTERTERHETLGQWRNRLGNAGFETVHLGSNAYKQASTL    579
Wheat_CAB51555      TDQVMSEVYLGRQICNVVACEGAERTERHETLGQWRNRLGNAGFETVHLGSNAYKQASTL    584
                    **********************:************* ***.**** **************

LpDELLA             LALFAGGDGYKVDEKEGCLTLGWHTRPLIATSAWRMAAAAAP   612
Rice_BAE96289       LALFAGGDGYRVEEKEGCLTLGWHTRPLIATSAWRVAAA---   625
Barley_AAL66734     LALFAGGDGYKVEEKEGCLTLGWHTRPLIATSAWRLAAP---   618
Wheat_CAB51555      LALFAGGDGYKVEEKEGCLTLGWHTRPLIATSAWRLAGP---   623
                    **********:*:**********************:*.
```

Supplementary Figure 2 ClustalO alignment of the deduced LpDELLA protein with DELLA proteins from *O. sativa* SLR1 (Acc. # BAE96289; 80% id.), *H. vulgare* SLN1 (Acc. # AAL66734; 84% id.), and *T. aestivum* RHT-D1a (Acc. # CAB51555; 85% id.). Identity (*), strongly similar (:), and weakly similar (.). Sequences with an underline correspond to conserved motifs.

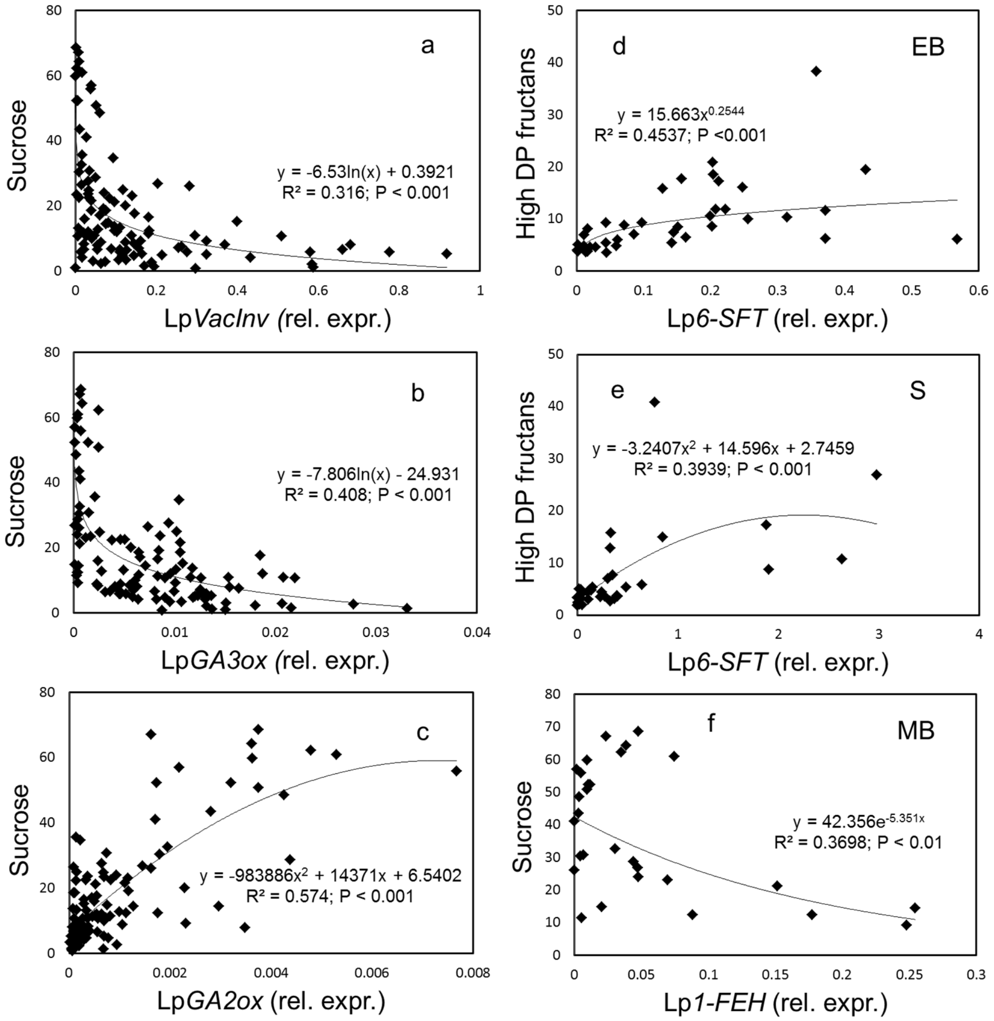

Supplementary Figure 3 Non-linear regression of transcript levels and carbohydrate content. (A) Lp*VacInv* vs. sucrose in all test tissues; (B) Lp*GA3ox* vs. sucrose in all tested tissues; (C) Lp*GA2ox* vs. sucrose in all tested tissues; (D) Lp*6-SFT* vs. high DP fructans in immature elongating blades; (E) Lp*6-SFT* vs. low DP fructans in mature sheaths; and (F) Lp*1-FEH* vs. sucrose in mature blades.
